# Are Family Physical Activity Habits Passed on to Their Children?

**DOI:** 10.3389/fpsyg.2021.741735

**Published:** 2021-09-06

**Authors:** Vinko Zovko, Sasa Djuric, Vedrana Sember, Gregor Jurak

**Affiliations:** ^1^Institute of Kinesiology, Faculty of Sport, University of Ljubljana, Ljubljana, Slovenia; ^2^Educational Unit for Sports, School of Economics and Business, University of Ljubljana, Ljubljana, Slovenia

**Keywords:** accelerometry, monitoring, MVPA, physical activity, sedentary behavior

## Abstract

Studies of the familial association of physical activity (PA) and sedentary behavior (SB) have increased in recent years. However, there is a lack of studies that have objectively examined the correlates between parents, grandparents, and childrens' PA. Therefore, the purpose of this study was to measure PA using accelerometers to determine the extent to which PA and SB correlate among parents, grandparents, and children. A sample of 169 children between 11 and 14 years (77 boys and 97 girls), 225 parents (98 males and 127 females), and 52 grandparents (16 males and 36 females) were recruited for the current study. Accelerometers RM42 (UKK Terveyspalvelut Oy, Tampere, Finland) were used to determine PA levels of children, parents, and grandparents. Epoch length was 6 s. Mothers' moderate-to-vigorous PA (MVPA) was associated with children's MVPA (*p* < 0.05). After adjusting for age, BMI (child), and educational status, the results remain the same. Results of linear regression analyses for boys' sedentary time showed that fathers' sedentary time was significantly associated with boys (*p* < 0.01), but not with girls. The association of grandmothers' and grandfathers' MVPA activity with that of children showed that grandparents' MVPA, when adjusted for age, BMI, and educational status, was not a significant predictor (*p* > 0.05) of children's MVPA (total sample). In contrast, grandfathers' sedentary behavior was a significant predictor (β = 0.269; *p* < 0.05) of children's sedentary behavior (total sample). The results of the current study suggest that parental involvement in PA, particularly by mothers, is important for children's PA and, accordingly, healthy outcomes.

## Introduction

Routines created in early childhood can strongly influence children's health. According to current guidelines (Tremblay et al., 2016; World Health Organization, 2020), which provide evidence-based recommendations for a healthy day (24 h), a combination of sleep, sedentary behaviors, light, moderate, and vigorous physical activity (PA) is essential for a healthy life.

Several studies showed a strong association between PA and a cardiometabolic risk profile in childhood (Andersen et al., 2006; Wittmeier et al., 2008; Steele et al., 2009; Ekelund et al., 2011) and later in adulthood (Kallio et al., 2021). Studies looking at factors that influence participation in PA in childhood have been the focus of scientific research for some time. There is a wide range of factors that have been investigated, including age, gender, socio-demographics, lifestyle and parental and peer influences as the most studied correlates (Craggs et al., 2011; Padulo et al., 2019).

There is strong evidence that parents' sociodemographic characteristics are associated with children's health (Dow and Rehkopf, 2010; Serbin et al., 2014). Parents serve as role models for their children and have a major influence on their PA (Birch and Davison, 2001; Jago et al., 2010; Lee et al., 2018). They encourage their children or instrumentally support their PA by taking them to events where they can be physically active (Gustafson and Rhodes, 2006).

Researchers have found that higher levels of parental education in the family environment are more relevant factors for more frequent PA in children and adolescents (Yang et al., 1996; Jiménez-Pavón et al., 2012; Vella et al., 2014). A review article that examined associations between PA in children and parents found conflicting results (Gustafson and Rhodes, 2006), most likely due to different methods of monitoring PA and the more frequent use of subjective rather than objective methods to determine PA (Gustafson and Rhodes, 2006; Jago et al., 2010). Most studies have used subjective methods to determine PA, particularly questionnaires, which are less reliable than objective methods. Studies using objective methods to determine PA most commonly use accelerometers for this purpose (Troiano et al., 2014), which provide an objective determination of an individual's PA based on objectively measured accelerations and using algorithms. Previous studies that have dealt with the determination of the PA of families based on accelerometers showed mixed results, especially considering gender (Hennessy et al., 2010; Fuemmeler et al., 2011; Garriguet et al., 2017; Song et al., 2017). Hennessy et al. (2010) found that parenting style and some parenting practices were related to children's PA. One study found a relationship only between PA levels of parents and boys, but not girls (Dozier et al., 2020). In addition, a longitudinal study showed that parents' PA was not related to children's PA (Anderssen et al., 2006). The reasons for these results could vary, from covariates included in the analysis to a number of days participants were observed.

Studies of parent-child correlates of PA using accelerometers have increased in recent years. However, to the authors' knowledge, no single study has examined objectively measured grandparent-child PA correlates. Considering that grandparents are often involved in children's lives through education and PA in some settings, it would be interesting to determine their influence on children's PA behaviors alongside that of parents. Therefore, the aim of this study was to investigate family-child PA associations using accelerometry.

## Materials and Methods

### Subjects

A sample of 174 children aged between 11 and 14 years of age (77 boys and 97 girls) and their families (225 parents and 52 grandparents) were recruited for the current study. The descriptive characteristics of the participants are presented in Table 1.

**Table 1:** Demographic characteristics of included participants.

	**Children** ***n*** **=** **174**		**Parents** ***n*** **=** **225**		**Grandparents** ***n*** **=** **52**	
	**Boys *n* = 77**	**Girls *n* = 97**	**Male *n* = 98**	**Female *n* = 127**	**Male *n* = 16**	**Female *n* = 36**
age (years)	12.3 ± 1.2	12.5 ± 1.5	44.5 ± 4.9	43.0 ± 6.0	68.2 ± 6.3	66.2 ± 6.6
height (cm)	160.3 ± 10.9	160.4 ± 8.5	178.8 ± 7.4	168.1 ± 11.5	175.7 ± 6.3	161.3 ± 5.6
weight (kg)	50.1 ± 12.6	50.4 ± 10.3	83.8 ± 14.0	67.4 ± 12.5	86.9 ± 16.6	70.8 ± 10.5
BMI (kg/m^2^)	19.5 ± 3.1	19.0 ± 3.1	26.2 ± 3.5	23.9 ± 4.2	28.2 ± 5.7	27.2 ± 4.9

*n, number of subjects; BMI, body mass index*.

The participants were primary school children from nine primary schools from urban and rural areas of Republic of Slovenia and their parents. The collection of participants was led by school coordinators who presented the research at parent meetings and collected signed consent forms from participants (for minor participants, they were signed by the parents). Due to the nature of the study, which requires consent from all family members, a voluntary response sample was used (Murairwa, 2015). The sample used for the analysis was designed based on the families of school-age children by including children between 11 and 14 years old and their parents. Siblings of the children who were no more than four years younger or older than them and their grandparents were also invited if they had regular contact with the child, i.e. at least twice a week.

Participants who were healthy and whose health was not affected by PA were included. The additional inclusion criterion for adults was that they were able to perform a 6-min walk test and run or walk the 600 m. Permission was obtained from the Faculty of Sport in Ljubljana in accordance with the Declaration of Helsinki (No: 6:2020-274). Data in present study were obtained within the project EUPASMOS No. 590662-EPP-1-2017-1-PT-SPO-SCP under the Erasmus+ Programme of European Union.

### Procedures

The measurements were performed in autumn 2018 (October to November). They took place at two time points, one week apart. At the first time point, the accelerometers were distributed to the participants and familiarized them with their use. The accelerometers were worn for a full week. Participants were instructed to wear the accelerometers continuously throughout the whole day (24-h) for consecutive seven days, except when bathing or swimming. At the second time point, participants returned the accelerometers.

The height and weight of the subjects were measured using an electronic scale and an anthropometer (Kern and Sohn GmbH, Balingen, Germany). PA was measured with an RM42 accelerometer (UKK RM42, UKK Terveyspalvelut Oy, Tampere, Finland), which is a triaxial accelerometer (data on duration and intensity of activity were recorded), and the data were later processed using the MAD approach (mean amplitude deviation). MAD values have been validated as indicators of energy expenditure during locomotion (Sievänen et al., 2014; Vähä-Ypyä et al., 2015a). Epoch length was 6 s and results are based on 1 min exponential moving average of epochwise MET-values. As recommended, the MAD values can be converted to metabolic equivalents (*MET* = 3.5 mL/kg/min oxygen consumption) for each epoch (Sievänen and Kujala, 2017).

The accelerometer was worn on an elastic band on the right side of the hip during the day and on the non-dominant arm at night for whole week. Several studies have shown high reliability of these devices based on coarse acceleration signals (89.2%) (Sievänen et al., 2014; Vähä-Ypyä et al., 2015b; Hukkanen et al., 2018).

Child-specific cut-points (Aittasalo et al., 2015) and adult cut-points (Vähä-Ypyä et al., 2015a) were used to categorize physical activity in minutes spent in the outcome categories of interest, namely sedentary (<1.5 METs) and moderate to vigorous PA (MVPA) (>3 METs). Because accelerometry data were collected continuously during the 24-h circadian cycle, several parameters describing patients' daily PA, SB, standing, and sleep profiles were assessed but excluded for regression analysis (Vähä-Ypyä et al., 2018). In addition, light METs (1.5–3 METs) were not included in the analyses.

### Statistical Analysis

A preliminary analysis of the data revealed that the PA variables were heavily skewed. Logarithmic transformations were therefore performed to normalize the data. Linear regression models were developed to assess the effects of parental PA on children PA. The analysis began with the following cross-sectional estimates:


(1) $$\begin{array}{l} \ln\left(MVPA\;child_{i}\right)=\beta_{0}+\beta_{1}\ln\left(MVPA\;parent_{i}\right)+\varepsilon_{i} \end{array}$$

(2) $$\begin{array}{l} \ln\left(SB\;child_{i}\right)=\beta_{0}+\beta_{1}\ln\left(SB\;parent_{i}\right)+\varepsilon_{i} \end{array}$$

where ln(*MVPA child*_*i*_) denotes the logarithm of the child's moderate and vigorous PA time, ln(*MVPA parent*_*i*_) represents the logarithm of the parent's moderate and vigorous PA time, ln(*SB child*_*i*_) is the logarithm of the child's sitting and laying time, while ln(*SB parent*_*i*_) represents the logarithm of the parent's sitting and laying time. In addition, adjusted models were estimated including covariates and potential confounders that could influence the relationships between the parent's and the child's PA, namely the child's age and BMI, as well as the parent's education level. Both unadjusted and adjusted models were estimated separately for boys and girls. Finally, the study estimated additional models to assess the relationship between grandparent's and grandchild's PA. Unfortunately, these models could not be estimated separately for boys and girls due to small sample size. All presented statistical analyses were conducted in Stata Statistical Software 2017: Release 15 (StataCorp LLC, Texas, US).

## Results

Table 2 shows the mean ± SD for minutes per day of light and MVPA, and the mean counts for SB and standing activities.

**Table 2:** Means and standard deviations for minutes per day of PA and SB.

	**Children**		**Parents**		**Grandparents**	
	**Boys**	**Girls**	**Male**	**Female**	**Male**	**Female**
SB (min·d^1^)	498.8 ± 72.2	504.3 ± 75.3	531.8 ± 105.0	497.8 ± 106.6	520.0 ± 149.2	489.8 ± 131.2
Standing (min·d^1^)	63.0 ± 36.7	78.5 ± 41.9	125.4 ± 61.1	142.1 ± 59.2	91.4 ± 31.4	130.2 ± 46.6
Light PA (min·d^1^)	221.2 ± 40.6	222.9 ± 45.2	258.4 ± 77.1	270.9 ± 82.4	215.2 ± 88.8	266.7 ± 72.9
MVPA (min·d^1^)	108.8 ± 38.5	78.5 ± 24.6	60.8 ± 54.1	47.7 ± 21.9	64.7 ± 38.4	44.5 ± 32.2

*PA, physical activity; SB, sedentary behavior*.

Results of the linear regression analyses represent the association of mothers' and fathers' PA with children's PA, adjusted for age, BMI (child), and educational status (Table 3).

**Table 3:** Linear regression analysis for variables predicting children's activity level.

	**MVPA**		**Sedentary behavior**	
	**B**	**R^**2**^**	**B**	**R^**2**^**
Boys				
Model 1				
Father	0.234	0.058	0.186	0.065
Mother	0.284^**^	0.114	0.048	0.004
Girls				
Model 1				
Father	0.149	0.032	0.138	0.039
Mother	0.166^*^	0.067	0.031	0.002
Boys				
Model 2				
Father	0.229	0.135	0.167	0.13
Mother	0.315^**^	0.181	0.044	0.197
Girls				
Model 2				
Father	0.161	0.054	0.158	0.273
Mother	0.178^*^	0.101	0.063	0.232

*MVPA, moderate-to-vigorous physical activity*.

** *p < 0.01*;

* *p < 0.05*.

*Model 1: Unadjusted*.

*Model 2: Adjusted for age, BMI and education*.

It was found that mothers' MVPA was significantly associated with children's MVPA (*p* < 0.05). The situation was the same after adjusting for age, BMI (child) and educational status (Model 2), only mothers' MVPA was a significant predictor of children's MVPA. Although mothers' and girls' PA were associated, the model explained only 10.1 % of the common variance. The situation was better for boys: after adjusting for age, BMI (child) and educational status, the model explained 18.1 %.

The results of linear regression analyses for SB (both models) showed that parents' SB was not significantly associated with neither boys nor with girls' SB (*p* > 0.05). In addition, girls' SB was associated with an increase in children's age (*p* < 0.01).

Linear regression analyses for the association of grandmothers' and grandfathers' MVPA activity with that of children (Model 1) showed that grandmothers' and grandfathers' MVPA was a significant predictor (Table 4) of children's MVPA (total sample). However, after adjusting for age, BMI, and educational status, both grandparents' MVPA was not a significant predictor (*p* > 0.05) of children's MVPA (total sample). In contrast, grandfathers' SB was a significant predictor (β = 0.269; *p* < 0.05) of children's SB (total sample), in a Model 2 that explained 35.8 % of the variance. It was also found that age was a significant predictor of the relationship between grandmothers and children (−0.075; *p* < 0.01).

**Table 4:** Linear regression analysis for variables predicting children's activity level.

	**MVPA**		**Sedentary behavior**	
	**B**	**R^**2**^**	**B**	**R^**2**^**
Total sample				
Model 1				
Grandfather	0.304^*^	0.189	0.278^*^	0.221
Grandmother	0.176^*^	0.083	0.021	0.001
Total sample				
Model 2				
Grandfather	0.182	0.415	0.269^*^	0.358
Grandmother	0.148	0.318	0.045	0.164

*MVPA, moderate-to-vigorous physical activity*.

* *p < 0.05*.

*Model 1: Unadjusted*.

*Model 2: Adjusted for age, BMI and education*.

## Discussion

The current study is important and unique because it contributes to the literature examining family-child associations in measuring PA with objective measures such as accelerometers. The main findings include the following: (i) mothers' MVPA is significantly associated with children's MVPA (*p* < 0.05); (ii) when adjusting for age, BMI (child) and educational status, only mother's MVPA showed as significant predictor of child's MVPA (10.1% of common variance for girls, *p* < 0.05; 18.1% of common variance for boys, *p* < 0.05) (iii) parental SB is not significantly associated with child's SB; (iv) grandparents' MVPA is a significant predictor of children's MVPA (*p* < 0.05); (v) only grandfathers' SB is a significant predictor (β = 0.269; *p* < 0.05) of children's SB, explaining 35.8 % of the common variance.

It was found that overall MVPA and SB were significantly associated between family members and children. However, the multivariate regression models showed that only mothers' MVPA was a significant predictor of children's MVPA, regardless of gender. The opposite results were obtained for the association with SB, which showed that fathers' and grandfathers' SB was a significant predictor of children's SB. The present results on family members' and children's PA patterns confirm the influence of parents' PA on children's PA and add important new insights into the grandparent-child relationship.

Previous findings on the PA levels association between parents and children showed different results, ranging from large correlations (Moore et al., 1991; Ruiz et al., 2011) to small (Ziviani et al., 2008; Østbye et al., 2013) or no significant correlations (Heitzler et al., 2010; Hennessy et al., 2010; Jago et al., 2010; Patnode et al., 2010; Dowda et al., 2011). In addition, a recent meta-analysis showed that parent-child PA correlates were greater with objective measurement than with self-reported questionnaires (Yao and Rhodes, 2015), resulting in more objective and reliable results of PA.

Moreover, studies have reported differences between boys and girls in both PA behaviors and influences on PA (Jago et al., 2010; Craggs et al., 2011; Fuemmeler et al., 2011; Cooper et al., 2015; Telford et al., 2016). Our findings are in line with a recent study that showed that maternal support had a significant influence on children's PA (Forthofer et al., 2016). However, this relationship changes from early childhood to adolescence, with fathers having the dominant influence on PA during preschool age, which changes later in life when mother takeover the dominance in PA (Abbott et al., 2016).

Although mothers' and girls' PA were associated, the model explained only 10.1 % of the variance. The situation was better for boys, when adjusted for age, the model explained 18.1 %. Although the current results did not find gender differences in PA, some studies have shown that boys are more active than girls at young adolescent age (Trost et al., 2002; Riddoch et al., 2004; Sember et al., 2020). A most recent study found a relationship among 8–12-year-old children only between PA levels of parents and boys, but not among girls (Dozier et al., 2020). However, in contrast to the aforementioned studies, a longitudinal study showed that PA of parents was not related to PA of children (Anderssen et al., 2006). In light of these findings, there is a need to understand how PA influences operate as children transit to adolescence. Therefore, it is clear that more longitudinal designs examining sex-specific parental influences on young children's PA are warranted.

Talking about the associations between parents and children regarding SB, some studies found higher rates of SB in children when parents that reported higher rates of screen time for their children (Mcguire et al., 2002; Kourlaba et al., 2009; Lee et al., 2010; Hoyos Cillero and Jago, 2011). However, when accelerometers were used to measure SB, no associations between parents' screen time and time spent in SB was found (King et al., 2011).

Currently, there is insufficient understanding of the pathways through which grandparents exert their influence on children's health and development outcomes (Sadruddin et al., 2019). This is the first study to investigate family-children relationships of objectively measured PA behaviors involving grandparents who live near their grandchildren and are involved in family daily activities/chores. However, after adjusting for age, BMI, and educational status, both grandparents' MVPA was not a significant predictor of children's MVPA. Possible reasons for the results regarding PA include several factors related to the costs, presence of grandchildren in the home, and cultural considerations (Kicklighter et al., 2007). In other societies, grandparents have a much greater influence on the family's daily routine than in ours. However, interestingly, grandfathers' SB was found to be a significant predictor of children's SB, explaining 35.8% of the variance. Unfortunately, it is difficult to compare our results with other studies because there are no studies that have investigated the association of grandparents' and children's PA. However, some studies have found negative associations between grandparent involvement and children's healthy eating habits (Pearce et al., 2010; Roberts and Pettigrew, 2010). In addition, one study found no relationship between grandparent involvement and children's PA behaviors (Pulgarón et al., 2013). Therefore, there is a need for future research on grandparent and child health.

## Considerations

The present study is the first to examine correlations between PA and SB in children, their parents and grandparents. Major strength of present study is the extension of previous findings regarding parental influence on PA and SB regarding the influence of grandparents on children's PA and SB. Based on the published literature, separate analysis of parental's PA and SB is significantly adding to the knowledge regarding parental influence on children's PA and SB. Previous findings concerning MVPA between parents, family members, and children suggest that MVPA is related, which should be used to promote and increase MVPA in children and their parents. There is a need for future studies to confirm our findings, as well as to expand this body of literature by examining the PA correlates of parents, family members, and their children. In addition, there is a need to conduct a longitudinal study examining the family PA correlates to determine how PA and SB change over the years.

To our knowledge, this is the first study examining objectively measured PA and SB of three-generation family. Nevertheless, some considerations and potential limitations have been acknowledged. First, the sample size for grandparents was small and diverse by gender (grandfathers *n* = 16; grandmothers *n* = 36), having a potential impact on the family correlates of PA and SB. In addition, a methodological limitations regarding the use of accelerometers should be mentioned: (i) although validated for measuring PA and SB, the present study did not not capture certain types of activities, such as light PA and very high PA; (ii) daily PA minutes (means and standard deviations) were very high, which may be a consequence of “cherry-picking,” where only the most active families decided to participate in the study; (iii) as in all PA studies, participants were aware that they were being monitored, which might have resulted in the changes of their habitual PA patterns; (iv) high measurement and equipment costs limited the availability of accelerometers and did not allow the measurement of PA of all included participants during the same week which means that the PA patterns could be affected by other factors such as different weather conditions; (v) MVPA and SB were measured for only one week, so the results cannot be interpreted as behavioral PA; (vi) biomechanical and physical factors could affect the results as increased mass give lower acceleration and the increased and/or different height of participants produces longer or shorter pendulum, resulting in potentially different measures od PA and SB; (vii) external factors could influence PA and SB: family problems, friendship problems, dietary habits and changes of environment/school, socioeconomic status, which have not been controlled for; (viii) this study was conducted in October to November, so the conclusions cannot be interpreted for a longer period of time, as previous studies have shown that older adults change their PA depending on the season (Padulo et al., 2018).

## Conclusion

The results of the current study suggest that parental involvement in PA, especially mothers, is significantly important for children's PA and, accordingly, their health outcomes. The discrepancy between mothers' and fathers' influence on the child PA is an interesting starting point for planning interventions that would encourage fathers to take on a greater role as promoters of PA. In addition, mothers' PA should be further encouraged and increased in order to increase PA in children. On the contrary, there is a need for additional support for grandfathers, considering that their sedentary behavior was a significant predictor of children's behavior. The present study is important because it makes a significant contribution to the study of family correlates in accelerometer-derived measures of physical activity.

## Data Availability Statement

The raw data supporting the conclusions of this article will be made available by the authors, without undue reservation.

## Ethics Statement

This study involved human participants. It was reviewed and approved by Ethics Committee of the Faculty of Sport, University of Ljubljana and was in accordance with the Declaration of Helsinki (No: 6:2020-274). Written informed consent to participate in this study was provided by the participants or parents/legal guardian for children involved in this study.

## Author Contributions

VZ, SD, VS, and GJ conceptualized the study design and recruited subjects into the study. GJ conducted the research. VS and VZ analyzed and interpreted the data. VZ drafted the manuscript. VS, SD, and GJ reviewed the manuscript. All authors have read and approved the final version of the manuscript.

## Conflict of Interest

The authors declare that the research was conducted in the absence of any commercial or financial relationships that could be construed as a potential conflict of interest.

## Publisher's Note

All claims expressed in this article are solely those of the authors and do not necessarily represent those of their affiliated organizations, or those of the publisher, the editors and the reviewers. Any product that may be evaluated in this article, or claim that may be made by its manufacturer, is not guaranteed or endorsed by the publisher.
