# Corrigendum: Are Family Physical Activity Habits Passed on to Their Children?

**DOI:** 10.3389/fpsyg.2022.824595

**Published:** 2022-03-08

**Authors:** Vinko Zovko, Sasa Djuric, Vedrana Sember, Gregor Jurak

**Affiliations:** ^1^Institute of Kinesiology, Faculty of Sport, University of Ljubljana, Ljubljana, Slovenia; ^2^Educational Unit for Sports, School of Economics and Business, University of Ljubljana, Ljubljana, Slovenia

**Keywords:** accelerometry, monitoring, MVPA, physical activity, sedentary behavior

In the original article, there was an error in the **Abstract** as published. “Epoch was set to 1 s” should have been “Epoch length was 6 s.” A correction has been made to the **Abstract** as below:

Studies of the familial association of physical activity (PA) and sedentary behavior (SB) have increased in recent years. However, there is a lack of studies that have objectively examined the correlates between parents, grandparents, and childrens' PA. Therefore, the purpose of this study was to measure PA using accelerometers to determine the extent to which PA and SB correlate among parents, grandparents, and children. A sample of 169 children between 11 and 14 years (77 boys and 97 girls), 225 parents (98 males and 127 females), and 52 grandparents (16 males and 36 females) were recruited for the current study. Accelerometers RM42 (UKK Terveyspalvelut Oy, Tampere, Finland) were used to determine PA levels of children, parents, and grandparents. Epoch length was 6 s. Mothers' moderate-to-vigorous PA (MVPA) was associated with children's MVPA (*p* < 0.05). After adjusting for age, BMI (child), and educational status, the results remain the same. Results of linear regression analyses for boys' sedentary time showed that fathers' sedentary time was significantly associated with boys (*p* < 0.01), but not with girls. The association of grandmothers' and grandfathers' MVPA activity with that of children showed that grandparents' MVPA, when adjusted for age, BMI, and educational status, was not a significant predictor (*p* > 0.05) of children's MVPA (total sample). In contrast, grandfathers' sedentary behavior was a significant predictor (β = 0.269; *p* < 0.05) of children's sedentary behavior (total sample). The results of the current study suggest that parental involvement in PA, particularly by mothers, is important for children's PA and, accordingly, healthy outcomes.

In the original article, there was an error in **Materials and Methods**, **Procedures**, Paragraph 2. “Signals were recorded using 1-s epoch.” should have been “Epoch length was 6 s and results are based on 1 min exponential moving average of epochwise MET-values.” A correction has been made to **Materials and Methods**, **Procedures**, Paragraph 2 as below:

The height and weight of the subjects were measured using an electronic scale and an anthropometer (Kern and Sohn GmbH, Balingen, Germany). PA was measured with an RM42 accelerometer (UKK RM42, UKK Terveyspalvelut Oy, Tampere, Finland), which is a triaxial accelerometer (data on duration and intensity of activity were recorded), and the data were later processed using the MAD approach (mean amplitude deviation). MAD values have been validated as indicators of energy expenditure during locomotion (Sievänen et al., 2014; Vähä-Ypyä et al., 2015a). Epoch length was 6 s and results are based on 1 min exponential moving average of epochwise MET-values. As recommended, the MAD values can be converted to metabolic equivalents (*MET* = 3.5 mL/kg/min oxygen consumption) for each epoch (Sievänen and Kujala, 2017).

In the original article, there was an error in **Materials and Methods**, **Procedures**, Paragraph 3. The second part of sentence 2 “compared to the double-labeled water method, which is considered the gold standard for determining physical activity according to objective criteria (Sirard and Pate, 2001)” was incorrect. The corrected **Materials and Methods**, **Procedures**, Paragraph 3 appears below:

The accelerometer was worn on an elastic band on the right side of the hip during the day and on the non-dominant arm at night for whole week. Several studies have shown high reliability of these devices based on coarse acceleration signals (89.2%) (Sievänen et al., 2014; Vähä-Ypyä et al., 2015b; Hukkanen et al., 2018).

In the original article there was also an error in **Materials and Methods**, **Procedures**, Paragraph 4. Vähä-Ypyä et al. (2018) and Aittasalo et al. (2015) were not cited in the article. Trost et al. (2002) was incorrectly cited in the article. The citations have now been amended in **Materials and Methods**, **Procedures**, Paragraph 4 below:

Child-specific cut-points (Aittasalo et al., 2015) and adult cut-points (Vähä-Ypyä et al., 2015a) were used to categorize physical activity in minutes spent in the outcome categories of interest, namely sedentary (<1.5 METs) and moderate to vigorous PA (MVPA) (>3 METs). Because accelerometry data were collected continuously during the 24-h circadian cycle, several parameters describing patients' daily PA, SB, standing, and sleep profiles were assessed but excluded for regression analysis (Vähä-Ypyä et al., 2018). In addition, light METs (1.5–3 METs) were not included in the analyses.

**Deleted References**

Freedson, P. S., Melanson, E., and Sirard, J. (1998). Calibration of the computer science and applications, inc. accelerometer. *Med. Sci. Sports Exerc*. 30, 777–781. doi: 10.1097/00005768-199805000-00021

Sirard, J. R., and Pate, R. R. (2001). Physical activity assessment in children and adolescents. *Sport. Med*. 31, 439–454. doi: 10.2165/00007256-200131060-00004

The authors apologize for this error and state that this does not change the scientific conclusions of the article in any way. The original article has been updated.

## Conflict of Interest

The authors declare that the research was conducted in the absence of any commercial or financial relationships that could be construed as a potential conflict of interest. The handling editor declared a past co-authorship with one of the authors VS.

## Publisher's Note

All claims expressed in this article are solely those of the authors and do not necessarily represent those of their affiliated organizations, or those of the publisher, the editors and the reviewers. Any product that may be evaluated in this article, or claim that may be made by its manufacturer, is not guaranteed or endorsed by the publisher.
